# Supplementary material for: The silent majority: The typical Canadian sex worker may not be who we think
Source: PLoS One. 2022 Nov 15;17(11):e0277550. doi: 10.1371/journal.pone.0277550 (PMC9665380; doi:10.1371/journal.pone.0277550)
Supplement: S1 Appendix — (DOCX) [file pone.0277550.s001.docx]

## S1 Appendix: Image validation

The image data was analyzed to find common images using the python *ImageHash* module [1–3]**.** In addition, images were processed using the python *mtcnn* module to detect faces [1,4,5]. Face detection helped to distinguish images with people from other types of graphics.

The image processing data was validated by visually inspecting samples of images. Python’s *ImageHash* implementation of the perceptual hash algorithm should identify images that are the same despite changes in scaling, cropping and other types of manipulation. To confirm that this was the case, generated hashes were tested using a sample of 3000 images from 1000 perceptual hashes. No errors were found even in images where faces were obscured or where images were partly covered in text.

The *mtcnn* library provides a confidence score of between 0 and 1 when it detects a face. The maximum confidence score for each image tested was stored with the image data. To verify that the maximum *mtcnn* confidence score was a valid indicator that that image contained a face, a sample of 4448 images was checked against the maximum confidence score for the images. Accuracy was maximized at 93.1% with a confidence score of 0.925 or higher (N=4130, true positives 761, true negatives 3085, false positives 146, false negatives 138, precision 0.85, recall 0.84, f score 0.84). Overall, 2893 (65.0%) of the images checked had no detectable face, 526 (11.8%) had a partially visible face and 1029 (23.1%) had a fully visible face.

Most images with faces in them appeared to be authentic, meaning they appeared to have been taken by the advertisers themselves. In a sample of 1029 unique face images based on phash values, 902 (87.7%) were deemed to be valid images with faces, 127 (12.3%) were deemed to be taken from other sources such as commercial pornography or advertising stock photos.

## Bibliography

1. Population Project. Pop Downloader. 2021. Available: https://gitlab.com/population.project.2021/pop-downloader-public

2. Khelifi F, Jiang J. Perceptual image hashing based on virtual watermark detection. Trans Image Process. 2010;19: 981–994. doi:10.1109/TIP.2009.2038637

3. Buchner J. ImageHash. 2020. Available: https://pypi.org/project/ImageHash/

4. Zhang K, Zhang Z, Li Z, Qiao Y. Joint face detection and alignment using multitask cascaded convolutional networks. IEEE Signal Process Lett. 2016;23: 1499–1503. doi:10.1109/LSP.2016.2603342

5. de Paz Centeno I. MTCNN. 2019. Available: https://pypi.org/project/mtcnn/
