# Supplementary material for: The silent majority: The typical Canadian sex worker may not be who we think
Source: PLoS One. 2022 Nov 15;17(11):e0277550. doi: 10.1371/journal.pone.0277550 (PMC9665380; doi:10.1371/journal.pone.0277550)
Supplement: S2 Appendix — (DOCX) [file pone.0277550.s002.docx]

## S2 Appendix: Comparison with the SPACES study

Based on private communication with the SPACES investigators, between November 1, 2014 and November 1, 2015 an estimated 109829 advertisers were active [1]. For the same period this study identifies 89522 advertisers (unscaled). The SPACES investigators did not attempt to account for advertisers changing contacts over time. The comparable scaled advertiser estimate for this period was 40225 (95% CI 39603-40892) as over a year many of the advertisers would likely have used multiple contacts.

In the SPACES data, 88134 primary contacts were identified (82772 phone numbers and 5362 emails). In the same time period this study identified 91185 primary contacts (84121 phone numbers and 7064 emails). However, some primary contacts used in the SPACES data were excluded in the dataset used here (2102 excluded contacts representing 1928 phone numbers and 174 emails). Furthermore, SPACES included 30.8% (N=93) of the primary contacts associated with advertisers judged in this study to be irrelevant (N=302) (see *P(a relevant)* in “Error estimates” above). SPACES identified an additional 16 phone numbers and 2 emails that were not found in the data used for this study..

The clustering method used may account for some of the difference between the unscaled contacts seen in this study and the SPACES advertisers. The contact clusters generated using DBSCAN only contained one instance of a primary contact per cluster; however, many of the SPACES contact clusters shared primary contacts with other clusters. For the period of 2014-2015 SPACES identified 11842 advertisers with multiple contacts in contrast to 6845 clusters found here using DBSCAN.

The SPACES investigators also used image identifiers associated with ads and ad text to distinguish advertisers. In fact, 46987 advertisers are identified using these alone. This is surprising given that the majority (~89%) of ads use some form of identifiable primary contact. The image identifiers used by the SPACES investigators to identify advertisers consist of strings in the form *SHA1-size*. Where *size* is the size of the image file in bytes and *SHA1* [63] is a hash of the image file contents similar to the perceptual hash described above. Unlike perceptual hashing, which tends to produce identical hashes for similar images, SHA1 hashes for similar images, even when the changes were small, could be substantially different, potentially hiding the relationships between images and inflating advertiser counts. Based on combinations of contacts and images alone, 80668 unique advertisers were identified in the SPACES data, indicating that many advertisers were subdivided based on a combination of images, primary contacts and differences in ad text.

## Bibliography

1. Atchison C, Burnett P. Spaces study private communication. 2021.
