# Supplementary material for: The silent majority: The typical Canadian sex worker may not be who we think
Source: PLoS One. 2022 Nov 15;17(11):e0277550. doi: 10.1371/journal.pone.0277550 (PMC9665380; doi:10.1371/journal.pone.0277550)
Supplement: S3 Appendix — (DOCX) [file pone.0277550.s003.docx]

## S3 Appendix: Multiple populations

Gender was detected based on the location of the ad on the advertising websites. Out of 167539 advertisers (unscaled), most identified as cis female (N=140791, 84.0%) . However, significant majorities identified as cis male (N=15302, 9.1%), female identified transgendered (N=3831, 2.3%) and male identified transgendered (N=35, 0.02%).

Note that unscaled advertiser counts will be different from the scaled worker counts described above and are used for purposes of comparison only. The actual number of workers, while related, is not the same as the number of advertisers.

Most collectives were gender-consistent (89.1%) but 4.2% were mixed. Of the collective advertisers, 6.8% did not have an identified gender. The most common mixed gender collective was cis male with cis female (2.8%) followed by cis female with transgendered (1.0%).

Site 3 metadata on self-identified ethnicity is summarized in Table 5. This compares data collected between November 1, 2014 and December 31, 2016 with data collected between October 1, 2021 and August 1, 2022. In 2014-2016 most Site 3 advertisers self-identified as white (53.0%) with “mixed” being the next most prevalent group at (17.8%). Asian advertisers were the third most prevalent group (13.9%). Proportions were compared between the 2014-2016 data collection period and the 2021-2022 data collection period using the R language *prop.test* function [1]. Between 2014-2016 and 2021-2022 the only ethnicity that did not change significantly was Indigenous (“Native”). Asian, White and Mixed categories all decreased significantly while Black, Canadian Born Chinese, Indo Canadian, Hispanic and Middle Eastern all increased significantly.

**Table 5: Unscaled counts of Site 3 advertisers by self-identified ethnicity.**

| **Ethnicity** | **Advertisers (2014-2016)** | **Chat ids (2021-2022)** | **Change*** |
| --- | --- | --- | --- |
| Asian | 1550 (13.9%) | 4393 (10.8%) | - |
| Black | 496 (4.4%) | 3285 (8.1%) | + |
| White | 5925 (53.0%) | 19810 (48.5%) | - |
| Canadian Born Chinese | 163 (1.5%) | 435 (2.4%) | + |
| Indo Canadian | 198 (1.8%) | 995 (3.1%) | + |
| Hispanic | 530 (4.7%) | 3857 (9.5%) | + |
| Middle Eastern | 189 (1.7%) | 1163 (2.9%) | + |
| Mixed | 1989 (17.8%) | 6397 (15.7%) | - |
| Native | 140 (1.3%) | 494 (1.2%) | = (p = 0.72) |

*Changes from 2014-2016 to 2021-2022: + significant increase (p < 0.001), - significant decrease, = no significant change.

## Bibliography

1. R Core Team. R: A Language and Environment for Statistical Computing. Vienna, Austria: R Foundation for Statistical Computing; 2021. Available: https://www.R-project.org/
