# Supplementary material for: The silent majority: The typical Canadian sex worker may not be who we think
Source: PLoS One. 2022 Nov 15;17(11):e0277550. doi: 10.1371/journal.pone.0277550 (PMC9665380; doi:10.1371/journal.pone.0277550)
Supplement: S4 Appendix — (DOCX) [file pone.0277550.s004.docx]

## S4 Appendix: Factors affecting advertiser longevity

In order to track advertising behavior, ad count and the first and last time ads containing advertiser contacts were recorded for each advertiser. To develop more insight into why advertisers may have continued advertising, metadata and post content were checked for demographic data such as advertised language, ethnicity, body type and gender. Also, a number of behavioral dimensions were tracked:

- Drug use (using the keywords “party friendly” or “420 friendly”).
- Willingness to be identified (if the advertiser had images showing a face).
- Service types (keywords “escort”, “massage”, “fetish”, “tantric”) based on where an ad was placed on the hosting site and, for “tantric”, ad content itself. Only Site 1 did not have categories for ads.
- Street-based workers based on ad keywords such as “car call”, “car date”, “blow n go”.
- Travel based on phrases commonly associated with travel such as “last day”, “travel”, “new in town”, “visiting” or ”just arrived” and, secondly, out of area phone numbers.
- Restrictions were tallied based on phrases found in ads starting with the word “no” (see “restrictions” in [1])

Overall, advertisers were visible for a mean 73.3 days (SD 151.8, median 14, IQR 1-58). Names were visible for a mean of 26.6 days (SD 71.6, median 2). Ads were visible for a mean of 10.4 days (SD 41.0, median 1).

The probability that any advertiser will be online for a specific number of days follows the power law formula

$p\left( days \right)=C*{days}^{-1.32}$ (8)

where

$1.32\approx\alpha=1+\frac{count\left( advertisers \right)}{\sum_{a\in advertisers} ln\left( days\left( a \right)/{0.5} \right)}$(9)

from the technique described in [2]. *p(days)* represents the probability that an advertiser had advertised for *days* days where *1 <= days* and *C* is an arbitrary constant. *count(advertisers)* is the total number of unscaled advertisers and *days(a)* is the number of days a given advertiser was online where *1 <= days(a)*.

*p(days)* was significantly positively correlated with the actual proportion of advertisers advertising for that number of days (pearson correlation 0.98, p < 0.001). Correlation was calculated using the R language *cor.test* function [3].

Variables such as gender, collective vs individual, travel etc. all affected advertiser longevity. However, overall for any given category, the dimension of collective versus individual significantly increased days online for every variable - defined as the time between the date of the first ad and the last day the last ad was seen. Z tests of individual versus collective were p < 0.001 overall and this was also true for all subcategories. Comparing medians, collective advertisers’ median days online were 6.5 times longer than individual advertisers overall. The category with the least difference between collective and individual advertisers was the “male” category with median 31 days and 13 days for collective and individual advertisers respectively. The category with the longest-duration advertisers was the “tantric” or “energy work” category with a median 124 days. Communities potentially affected by social stigma such as fetish, “big beautiful woman” (BBW), transgendered and street involved tended to advertise for longer periods as well. See S2 File in the supplemental materials for a detailed breakdown by category.

A similar pattern is seen for the total number of ads posted (see S3 File in the supplemental materials). Median ads per advertiser during 2014-2016 were 2 overall (median 2 for individuals and 5 for collective advertisers).

Out of 167539 advertisers, 59491 (35.5%, unscaled counts) only advertised once. These one-off ads were live for a median of 1 day (average 11.5 days, standard deviation: 38.0 days). Image reuse was common among these advertisers with 44454 (74.7%) of all single ad advertisers reusing images from other advertisers.

The qualitative literature discusses workers periodically quitting the business [4–7]. In the advertising data, among advertisers only using a single name who had been online for at least a year this was common: 798 (72.7%) of the 1098 who matched these criteria took at least one break from advertising greater than one week. The median break greater than one week was 30 days for long term individual advertisers. We should keep in mind that not advertising online does not necessarily mean that the advertiser is inactive. Advertising for more than a year was uncommon. The unscaled count of advertisers active for at least two years was 2429 (1.4%) and 9882 (5.9%) for advertisers active for at least one year.

What workers charged per hour did not have a significant effect with respect to days online. However, providing any price information at all was significant. Provided hourly rates were classified based on quartiles (first quartile: $160/hr, median: $200/hr, third quartile: $250/hr): 0 no data, 1 up to first quartile, 2 between first quartile and median, 3 between median and third quartile, 4 greater than third quartile. Table 6 shows the distribution of advertisers by rate. A kruskal-wallis test computed between categories 1, 2, 3 and 4 was not significant (chi-squared = 6.0363, df = 3, p-value = 0.1099) [3]. However, a kruskal-wallis test including the no data category was significant (chi-squared = 4760.4, df = 4, p-value < 2.2e-16). Tukey’s HSD test showed significant differences (p < 0.001) between the no data category and all other categories. Tests were done using R [“id-vars/priceclass.R” in 1,3].

**Table 6: Unscaled advertiser counts by advertised hourly rate.**

| **Price class** | **Advertisers** | **Name counts** |
| --- | --- | --- |
| No data | 155939 | 208876 |
| $160/hr or less | 2608 | 8349 |
| $161 to $200/hr | 3316 | 10109 |
| $201 to $250/hr | 2857 | 9439 |
| $250/hr or more | 2819 | 8101 |

Advertisers in any category showed a wide variation in advertising behavior, with large skewness in the distributions relating to both days online (skewness 3.1) and ads posted (skewness 104.6). The wide spectrum of advertising behavior was likely affected by the intersection of advertising venue, demographic variables, types of services offered and gender self-identity.

## Bibliography

1. Population Project. Pop Downloader. 2021. Available: https://gitlab.com/population.project.2021/pop-downloader-public

2. Clauset A, Shalizi CR, Newman MEJ. Power-Law Distributions in Empirical Data. SIAM review. 2009;51: 661–703.

3. R Core Team. R: A Language and Environment for Statistical Computing. Vienna, Austria: R Foundation for Statistical Computing; 2021. Available: https://www.R-project.org/

4. Prostitution Law Review Committee. Report of the Prostitution Law Review Committee on the Operation of the Prostitution Reform Act 2003. Government of New Zealand; 2008 p. 177. Available: https://prostitutescollective.net/wp-content/uploads/2016/10/report-of-the-nz-prostitution-law-committee-2008.pdf

5. Benoit C, Millar A. Dispelling Myths and Understanding Realities: Working Conditions, Health Status, and Exiting Experiences of Sex Workers. 2001 p. 133.

6. Jeffrey LA, MacDonald G. “It’s the Money, Honey”: The Economy of Sex Work in the Maritimes*. The Canadian Review of Sociology and Anthropology. 2006;43: 313–327.

7. O’Doherty T. Off-street Commercial Sex: An exploratory Study. Thesis (M.A.), Simon Fraser University. 2007. Available: http://summit.sfu.ca/item/8064
