# Supplementary material for: The silent majority: The typical Canadian sex worker may not be who we think
Source: PLoS One. 2022 Nov 15;17(11):e0277550. doi: 10.1371/journal.pone.0277550 (PMC9665380; doi:10.1371/journal.pone.0277550)
Supplement: Attachment — Submitted filename: Response to Reviewers.docx [file pone.0277550.s009.docx]

**[Responses in bold below]**

PONE-D-22-00964R1

The silent majority: the typical Canadian sex worker may not be who we think

PLOS ONE

Dear ~~Dr.~~ Kennedy,

Thank you for submitting your manuscript to PLOS ONE. After careful consideration, we feel that it has merit but does not fully meet PLOS ONE’s publication criteria as it currently stands. Therefore, we invite you to submit a revised version of the manuscript that addresses the points raised during the review process.

Please submit your revised manuscript by Dec 01 2022 11:59PM. If you will need more time than this to complete your revisions, please reply to this message or contact the journal office at plosone@plos.org. When you're ready to submit your revision, log on to https://www.editorialmanager.com/pone/ and select the 'Submissions Needing Revision' folder to locate your manuscript file.

Please include the following items when submitting your revised manuscript:

A rebuttal letter that responds to each point raised by the academic editor and reviewer(s). You should upload this letter as a separate file labeled 'Response to Reviewers'.

A marked-up copy of your manuscript that highlights changes made to the original version. You should upload this as a separate file labeled 'Revised Manuscript with Track Changes'.

An unmarked version of your revised paper without tracked changes. You should upload this as a separate file labeled 'Manuscript'.

If you would like to make changes to your financial disclosure, please include your updated statement in your cover letter. Guidelines for resubmitting your figure files are available below the reviewer comments at the end of this letter.

If applicable, we recommend that you deposit your laboratory protocols in protocols.io to enhance the reproducibility of your results. Protocols.io assigns your protocol its own identifier (DOI) so that it can be cited independently in the future. For instructions see: https://journals.plos.org/plosone/s/submission-guidelines#loc-laboratory-protocols. Additionally, PLOS ONE offers an option for publishing peer-reviewed Lab Protocol articles, which describe protocols hosted on protocols.io. Read more information on sharing protocols at https://plos.org/protocols?utm_medium=editorial-email&utm_source=authorletters&utm_campaign=protocols.

We look forward to receiving your revised manuscript.

Kind regards,

Hamid Sharifi

Academic Editor

PLOS ONE

Journal Requirements:

Please review your reference list to ensure that it is complete and correct. If you have cited papers that have been retracted, please include the rationale for doing so in the manuscript text, or remove these references and replace them with relevant current references. Any changes to the reference list should be mentioned in the rebuttal letter that accompanies your revised manuscript. If you need to cite a retracted article, indicate the article’s retracted status in the References list and also include a citation and full reference for the retraction notice.

- **All references were checked for retractions. No retractions were found as of 2022-10-17.**

[Note: HTML markup is below. Please do not edit.]

Reviewers' comments:

Reviewer's Responses to Questions

Comments to the Author

1. If the authors have adequately addressed your comments raised in a previous round of review and you feel that this manuscript is now acceptable for publication, you may indicate that here to bypass the “Comments to the Author” section, enter your conflict of interest statement in the “Confidential to Editor” section, and submit your "Accept" recommendation.

Reviewer #1: All comments have been addressed

Reviewer #2: All comments have been addressed

2. Is the manuscript technically sound, and do the data support the conclusions?

The manuscript must describe a technically sound piece of scientific research with data that supports the conclusions. Experiments must have been conducted rigorously, with appropriate controls, replication, and sample sizes. The conclusions must be drawn appropriately based on the data presented.

Reviewer #1: Yes

Reviewer #2: Yes

3. Has the statistical analysis been performed appropriately and rigorously?

Reviewer #1: I Don't Know

Reviewer #2: Yes

4. Have the authors made all data underlying the findings in their manuscript fully available?

The PLOS Data policy requires authors to make all data underlying the findings described in their manuscript fully available without restriction, with rare exception (please refer to the Data Availability Statement in the manuscript PDF file). The data should be provided as part of the manuscript or its supporting information, or deposited to a public repository. For example, in addition to summary statistics, the data points behind means, medians and variance measures should be available. If there are restrictions on publicly sharing data—e.g. participant privacy or use of data from a third party—those must be specified.

Reviewer #1: Yes

Reviewer #2: Yes

5. Is the manuscript presented in an intelligible fashion and written in standard English?

PLOS ONE does not copyedit accepted manuscripts, so the language in submitted articles must be clear, correct, and unambiguous. Any typographical or grammatical errors should be corrected at revision, so please note any specific errors here.

Reviewer #1: Yes

Reviewer #2: Yes

6. Review Comments to the Author

Please use the space provided to explain your answers to the questions above. You may also include additional comments for the author, including concerns about dual publication, research ethics, or publication ethics. (Please upload your review as an attachment if it exceeds 20,000 characters)

Reviewer #1: The authors have adequately addressed my comments raised in the previous round of review.

However, the revised manuscript has some new minor issues that I believe the authors need to address. These issues being minor however only entail minor revision of the manuscript.

1. The sentence starting in line 109 that begins with "Issues with these other source ....." could be better moved to the discussion section.

- **This has been moved to the Discussion > Limitations section paragraph 2.**

2. In Table 3 (from line 349), the authors present p values less than 0.001 as decimal numerals. However, the standard convention is for such p values to be expressed as p<0.001 regardless of the actual decimal numeral. I would therefore suggest that the authors consider following the standard convention and present all the p values that are less than 0.001 as p<0.001.

- **These have been changed to use the convention.**

3. In lines 388, 390 and 427, the authors present Odds Ratios (ORs) without the corresponding confidence intervals (CIs). I would suggest that they consider presenting all ORs with their corresponding CIs as per standard convention.

- **The main reason for the calculation was two-fold:**
  a. **Show that just using advertisers as a measure of collective vs individual can be misleading.**
  b. **Show that, while the Argento et al. sample was different on some measures, it was comparable to the advertising data on the collective vs individual dimension when considering indoor workers.**
- **The comparisons were redone using the R prop.test function. The text has been updated to reflect the results.**
- **Previously, the odds ratio was calculated by dividing the probability of an advertiser or name being found in a collective context with that of the same being found in an individual context. For example, for advertisers this would be:**

$Odds Ratio=\frac{p(Advertiser is collective)}{p(Advertiser is individual)}=\frac{\frac{{Advertisers}_{collective}}{Advertisers}}{\frac{{Advertisers}_{individual}}{Advertisers}}=\frac{{Advertisers}_{collective}}{{Advertisers}_{individual}}$

**As these are simple frequency counts, there is no confidence interval. In retrospect, prop.test seems to be more informative.**

Reviewer #2: Comments and questions raised in the previous revision have been incorporated or clarified.

The methodology is clear although a bit extensive.

- **Because this is the first time this has been attempted, the methods are necessarily more detailed. In the future, if related work is being described, it is hoped that the Methods in this paper can be used as a reference.**
